# Virtual versus Face-to-Face Cognitive Behavioral Treatment of Depression: Meta-Analytic Test of a Noninferiority Hypothesis and Men's Mental Health Inequities

**DOI:** 10.1155/2022/2972219

**Published:** 2022-06-02

**Authors:** Carly M. Charron, Kevin M. Gorey

**Affiliations:** University of Windsor, School of Social Work, 167 Ferry Street, Windsor, ON, Canada N9A 0C5

## Abstract

Global rates of depression have increased significantly since the beginning of the COVID-19 pandemic. It is unclear how the recent shift of many mental health services to virtual platforms has impacted service users, especially for the male population which are significantly more likely to complete suicide than women. This paper presents the findings of a rapid meta-analytic research synthesis of 17 randomized controlled trials on the relative efficacy of virtual versus traditional face-to-face cognitive behavioral therapy (CBT) in mitigating symptoms of depression. Participants' aggregated depression scores were compared upon completion of the therapy (posttest) and longest follow-up measurement. The results supported the noninferiority hypothesis indicating that the two modes of CBT delivery are equally efficacious, but the results proved to be significantly heterogeneous indicating the presence of moderating effects. Indirect suggestive evidence was found to support moderation by gender; that is, depressed males may benefit more from virtual CBT. Perhaps, this field's most telling descriptive finding was that boys/men have been grossly underrepresented in its trials. Future trials ought to oversample those who have been at this field's margins to advance the next generation of knowledge, allowing us to best serve people of all genders, those who live in poverty, Indigenous, Black, and other Peoples of Colour, as well as any others at risk of being marginalized or oppressed in contemporary mental health care systems.

## 1. Background

### 1.1. Depression and the COVID-19 Pandemic

According to the World Health Organization [1], the global prevalence of depression and anxiety has increased by 25% in the first year of the COVID-19 pandemic. Several factors have been found to be positively associated with depression during the pandemic, such as health-related vulnerabilities, poor socioeconomic characteristics (e.g., income difficulties, low family income, and reliance on financial support), preexisting medical conditions, experience of psychological and/or physical abuse, loneliness and social isolation, and type of work [2–5]. With the pandemic increasing the risk of developing or exacerbating depressive symptoms [2], it is essential to ensure that individuals have access to mental health support worldwide. Unfortunately, the conditions of COVID-19 have exacerbated preexisting mental health care access problems due to safety precautions and other limitations to in-person care [6–8]. To increase public safety and abide by social distancing policies, there has been a widespread shift to Internet-based and related telehealth methods of treatment since the beginning of 2020 [9–12]. Therefore, it is important to study the efficacy of offering existing treatment modalities (e.g., cognitive behavioral therapy) through virtual platforms.

### 1.2. Cognitive Behavioral Therapy

Cognitive behavioral therapy (CBT) is one of the most commonly researched and well-supported treatments for depression [13, 14]. CBT was originally developed for people with depression by Aaron Beck [15]. In the cognitive behavioral model of therapy, Beck identifies a connection between thoughts, emotions, and behaviour and argues that the way individuals perceive a situation will ultimately affect their subsequent reaction [16]. Therefore, therapists who practice CBT provide psychoeducation and seek to challenge biased or maladaptive cognitions in order to influence emotions and behaviour. Due to the structured and time-sensitive nature of CBT, it is ideal for individuals who are unable to commit to long-term therapy approaches or during times of natural disaster when limited resources need to be accessed by larger proportions of the population.

While strong evidence for the use of cognitive behavioral therapy (CBT) to treat depression was found in a review of 106 meta-analyses published between 2000 and 2012 [14], another meta-analysis found that dropout rates from CBT programs were significantly higher in virtual or e-therapy conditions than in treatments provided face-to-face (F2F). Such noncompletion of CBT was more common among people experiencing depression than among those with other mental health challenges [17]. Expeditious advancement of our understandings about the predictors and moderators of the effectiveness of such Internet-based treatments is needed now and likely will still be needed well into the pandemic's aftermath. Most critically, we need to better understand which groups of people might benefit most from virtual mental health care and which groups of people, if any, might be disadvantaged by it [18].

### 1.3. Men's Mental Health Disparities

One group that needs to be considered during the pandemic is men who may be at increased risk of depression. While men are not typically viewed as a disadvantaged group, research suggests otherwise in the realm of mental health. For example, men have historically underutilized both physical and mental health services. Scholars have theorized that this phenomenon may be due to a mismatch between masculine gender socialization/norms and the willingness to seek professional help, especially for mental health problems such as depression [19, 20]. This gender divide on mental health care access is probably intimately related to similar gender divides on other important outcomes. For example, the age-standardized global suicide completion rate is 1.8 times higher in men than in women [21]. According to the World Health Organization [21], female suicide rates are higher than male rates in only five of the 195 countries across the globe (Bangladesh, China, Lesotho, Morocco, and Myanmar). This substantial gender divide is curious since major depression is said to underlie more than half of suicides, yet depression is more commonly diagnosed in women [22]. Evidence suggests this diagnostic discrepancy remains even when mental health status and health care visit frequency are accounted for [23]. In addition, when compared within their respective genders, higher percentages of depressed women tend to seek help compared to depressed males; one large European study estimated help-seeking rates to be 59% compared to only 48% of depressed women and men, respectively [24].

Exploring gender differences on this horribly tragic outcome through treatment outcomes for depression is, therefore, clearly of great human and policy significance. Recent mental health advocacy movements for men have produced male-specific counselling guidelines that have been controversial and of little apparent avail thus far [25]. Despite barriers to mental health service access, recent evidence indicates that a large proportion of men *want* to seek help; in a cross-sectional survey with 778 males who self-reported a mental health concern, 65% reported a desire to seek treatment [26]. Decreasing barriers for men is therefore essential since CBT has been found to be effective in treating both men and women with depression [13, 27, 28]. While such evidence exists, little research has focused explicitly on men's experiences with CBT, especially within a virtual environment.

In the past, it has been suggested that altering therapeutic settings may be an effective way to engage men [29]. In support of this, one Australian study found an inverse relationship between suicidality and willingness to accept F2F counselling support; instead, a preference for online support in the face of suicidal thoughts was reported [30]. Perhaps, this suggests a timely means of facilitating mental health care access among depressed men by expanding the provision of virtual treatments. If, however, men do begin accessing therapy as a result of the COVID-19 pandemic's shift to virtual platforms, it is essential to measure its efficacy in order to ensure effective treatment for men.

### 1.4. Current Research State of Virtual CBT Efficacy

As previously noted, much strong, trial-based synthetic evidence in support of traditional, F2F CBT for depression has long existed [14]. Similar evidence to inform online or virtual CBT has also been developing over the past decade [31–34]. For example, these four previous systematic reviews and/or meta-analyses that overviewed nearly 50 randomized controlled trials (RCT) found consistent support for the overall effectiveness of virtual CBT with subclinical or clinically depressed people from childhood to older adulthood. However, most typically, these trials used nonactive, waiting list control groups. So, though we presently have quite strong evidence on the overall effectiveness of both virtual and F2F CBT, we have little evidence about their relative effectiveness, especially with specific populations such as men with depression.

Such questions as they relate to depression and other health challenges have most assuredly been much on the minds of practitioners and decision-makers as many have essentially been forced to switch to virtual treatments. The COVID-19 pandemic notwithstanding such seems quite important knowledge, knowledge that could be gleaned by a synthesis of noninferiority trials, that is, trials that directly compared virtual CBT with F2F CBT. This meta-analysis of noninferiority trials embedded within a rapid systematic review is our response. Finally, this field's previous reviewers have not yet tested the moderating influence of gender. Our hypotheses were as follows: (1) virtual CBT is not inferior to F2F CBT in the treatment of depression, (2) among depressed men, virtual CBT is more effective than F2F CBT.

## 2. Methods

This unfunded rapid systematic review and meta-analysis was also temporally constrained by the COVID-19 pandemic, our aim being to get the most recent and strongest evidence into the field as quickly as possible [35, 36]. The methods used were similar to those of our previous research [37], but this project involved a wider scope to allow for analysis of potential gender-based inequities in CBT research.

### 2.1. Sampling

To guard against publication bias, both peer-reviewed and unpublished, “grey” literature were included in the sampling frame [38, 39]. The following research literature databases were searched from inception until January 24, 2021: *Cochrane Central Register of Controlled Trials*, *PubMed/Medline*, *PsycINFO*, *Social Work Abstracts*, *CINAHL Complete*, *ProQuest Social Services Abstracts*, *Social Science Citation Index (Conference Proceedings)*, *ProQuest Dissertations and Theses*, the *Web of Science Conference Proceedings Index*, and *Google Scholar.* Article titles and abstracts were searched using the following broad keywords as criteria: (“cognitive behav∗ therapy” or “cognitive-behav∗ therapy” or CBT or iCBT or i-CBT or tCBT or t-CBT) and depress∗ and (virtual or online or Internet∗ or computer∗ or telephone or telemed∗ or telehealth or ehealth or e-health) and (RCT or “randomized controlled trial” or random or control∗ or trial or experiment). Additionally, studies had to meet the following criteria to be included in the meta-analysis: (1) participants had symptoms of depression or had been diagnosed with a major depressive disorder, (2) virtual CBT was assessed with a RCT, (3) control groups were largely similar to treatment groups, except that their CBT programs were provided F2F, and (4) written in English. RCTs that employed nonactive waiting list control groups or alternative, non-CBT control groups were excluded. Special populations such as those with serious comorbidities or dual diagnoses such as depression associated with pregnancy, cancer, or multiple sclerosis were also excluded.

**Figure 1:**
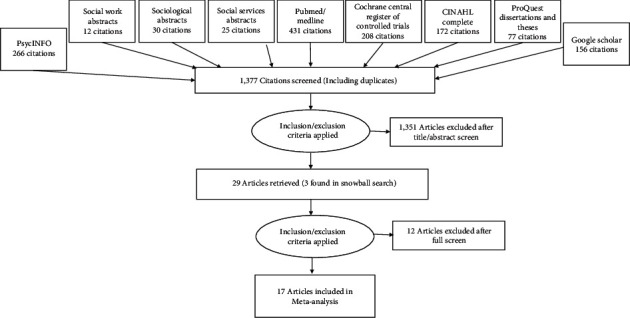
PRISMA diagram of literature search results.

Both reviewers searched for articles using these criteria, the breakdown of which is displayed in the PRISMA diagram in Figure 1 [40, 41]. Initial searches resulted in 1,377 potentially duplicated articles. After applying inclusion/exclusion criteria to the titles and abstracts, 1351 articles were excluded for their conceptual or empirical irrelevance, leaving 26 unique articles. Full manuscripts of these studies were then independently assessed by the two reviewers, achieving 83.3% agreement on ultimate study inclusion in this meta-analysis. Consensus or 100% agreement on inclusion of 14 independent RCTs was reached with discussion. Then, three additional independent studies were identified using such snowball search strategies as searching the bibliographies of the already selected studies, as well as searching for more eligible studies by their first, anchor, and corresponding authors' names. The reviewers agreed on the inclusion of three more RCTs, which resulted in a final meta-analytic sample of 17 RCTs. They are indicated with an asterisk in the references list.

### 2.2. Meta-Analytic Plan

The primary studies' diverse statistical outcomes were converted to Cohen's *d*-index to enable synthetic comparisons with a common effect size metric [42, 43]. It is the standardized mean difference between the treatment (virtual CBT) and control group (F2F CBT) at posttest or longer-term follow-up: *d* = M_vCBT_–M_F2FCBT_/((SD_vCBT_ + SD_F2FCBT_)/2). Though this noninferiority analysis hypothesizes between-group equivalence or the null value of *d* = 0.00, that is, that virtual CBT is not inferior to F2F CBT in psychotherapeutic work with depressed people, to track effect directions, ds were recorded as positive or negative to indicate if virtual or F2F CBT interventions led to greater decreases in depressive symptoms, respectively. To ensure that studies with larger samples had more influence in the pooled meta-analysis than less precise studies with smaller samples, random effects were weighted by their inverse variances ([44, 45]). Finally, the statistical significance of pooled meta-analytic findings was estimated with 95% confidence intervals (CI), two-tailed statistical significance at *α* < .05 being indicated when the CI does not include the null value of *d*_pooled_ = 0.00. All other statistical decisions were made at the two-tailed *α* < .05 criterion.

Each study contributed up to two data points for meta-analysis, one each for separate meta-analyses at immediate posttest or post-CBT intervention (16 RCTs) and for the longest follow-up assessment (11 RCTs). If a primary study provided multiple outcomes, typically standardized measures of depression, they were sample-weighted and pooled so that each study would contribute one data point for each of the meta-analytic hypothesis tests at posttest and follow-up. Then, the *d*_pooled_ distributions at posttest and follow-up were each tested for heterogeneity with Cochran's *Q*_t_ and accompanying *I*^2^ statistics [43, 46]. The resulting chi-square (*χ*^2^) distribution tests whether the variability of effects was greater than could be explained by sampling error alone, and *I*^2^ estimates the proportion of that variability that is likely explainable by real study differences (e.g., differences in their participants, interventions, contexts, or study designs) and not merely by random sampling error. Assuming significant heterogeneity, we tested the potential moderation of effects at posttest and follow-up by gender (relative greater [or lesser] study sample representation of men) with Cochran's *Q*_b_ statistic. It is essentially the meta-analytic version of a *t*-test and also follows a chi-square (*χ*^2^) distribution. Finally, all other personal and contextual study characteristics were extracted independently by both reviewers (initially 95.6% agreement with 100% agreement after discussion); their potential moderating influences were explored. The two meta-analytic runs were accomplished with version 3 of Comprehensive Meta-Analysis [47] and cross-validated by two analysts.

#### 2.2.1. Practical Significance Assessment

To allow for more practically interpreted statistics, weight *d*-indexes were converted to *U*_3_ statistics [42]. *U*_3_ critically compares all of the participants' scores in the “treated” group with the most typical or median score in the control group. By doing this, Cohen's *U*_3_ statistic tends to emphasize people, rather than statistics. For example, in this meta-analytic context, a *U*_3_ of 75% would have the following meaning: three-quarters, 75%, or 15 out of every 20 *people* in the virtual CBT treatment group scored lower at posttest on a standardized measure of depression than did the *typical person* in the F2F CBT control group.

## 3. Results

### 3.1. Sample Description

#### 3.1.1. Study Contexts and Participants

Descriptive characteristics of the 17 studies are displayed in Table 1. Sixteen RCTs are represented in total (one trial's results at posttest and follow-up were reported in two separate articles). The articles were published between 2003 and 2019 and accomplished in six countries: USA (7 studies), Europe (7; Netherlands, Sweden, or Switzerland), and Australia or New Zealand (3). The 16 immediate posttest assessments ranged from three to 20 weeks (median = 8 weeks) and the 11 follow-up assessments ranged from three months to three years (median = 6 months). As for the study participants, all age groups were represented from childhood (1 study) and adolescence (2) through emergent young adulthood (2) to older adulthood (3). The majority (9 studies) studied general adult samples 18 years of age or older. And there was a near equivalent mix of subclinical samples presenting with symptoms of depression (9 studies) and clinical or diagnosed samples, principally diagnosed with a major depressive disorder (8). Perhaps of most hypothetical importance, this synthetic sample's gender distribution was telling. The aggregated sample included 2,292 participants, only 615 or an approximate quarter of whom were boys or men (26.8%). No trials provided a nonbinary gender category in their demographic report of participants. Finally, half of the trials could be fairly categorized as racially inclusive, clearly overrepresenting certain racialized, ethnic, or cultural minority group members, while the others either did not report such racialized descriptions or predominantly studied non-Hispanic white people. Insufficient information was reported on other socioeconomic indicators to include them as descriptors or moderators.

#### 3.1.2. Study Designs and Interventions

Study samples ranged from 11 to 629 participants (median = 101), nearly one of every five of whom were lost to follow-up (17.6%). Such losses, however, did not differ significantly in aggregate between the virtual and F2F study groups. And most often analyses used intention to treat samples (11 studies), the others per protocol or completer samples. In the few instances where both were reported, we used study findings that were based upon the more conservative, intention to treat analyses. All of the studies used common and validated, standardized measures of depression. As for the CBT interventions, all of the trials had a F2F control condition. But the virtual CBT conditions varied with nine conducted online and four conducted over the phone, while the others were a combination or blending of these virtual intervention technological techniques. Finally, with a few extremes at either end of the distributions, the intervention's intensities and durations were fairly homogeneous. Ranging from 5 to 20 sessions over 3 to 20 weeks, the virtual and F2F CBT intervention programs clustered around 10 CBT sessions provided over 8 weeks.

#### 3.1.3. Adjunct, Preexperimental Descriptions

The results of preexperimental pooled analyses are displayed in the bottom of Table 1. There, the observed within-group improvement rates are sample-weighted, aggregated within the virtual and F2F CBT study groups, and compared. Both study groups improved significantly: virtual CBT *d*_pooled_ 1.35 (95% CI 1.25, 1.45) and F2F CBT *d*_pooled_ = 1.13 (95% CI 1.03, 1.23). Beyond mere statistical significance though, both were indicative of practical significance as well, demonstrating substantial reductions in depression symptoms from pretest to immediate posttest. The respective virtual and F2F study group's *U*_3_ statistics of 91.1 and 87.0 both similarly suggested the following. That is, that approximately nine of every 10 of their depressed study participants scored lower on standardized measures of depression at posttest that they themselves typically did at pretest.

### 3.2. Meta-Analytic Findings

#### 3.2.1. Main Interventive Effects at Posttest and Follow-Up

**Figure 2:**
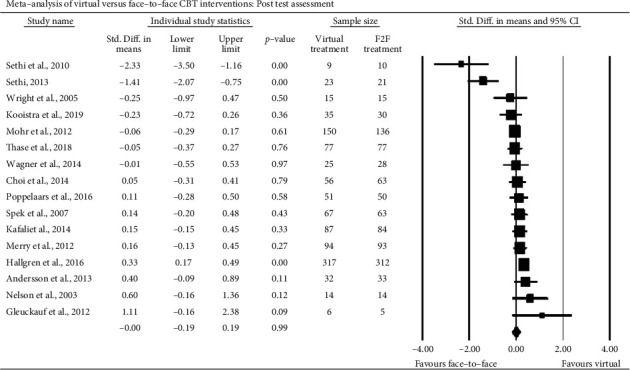
Critical posttest comparisons of virtual and face-to-face CBT.

The 16 study effects at immediate posttest are displayed from the smallest (*d* = −2.33) to largest (*d* = 1.11) point estimates in the, respective, top to bottom of Figure 2. One will first note that the most extreme standardized mean differences were associated with extremely small trials, and next, that 14 of the RCT findings were null. Furthermore, and in strong support of the main meta-analytic hypothesis that virtual CBT is not inferior to F2F CBT in work with depressed people, the synthetic estimate was exactly null and quite precise; *d*_pooled_ = −0.00 (95% CI -0.19, 0.19). Furthermore, the distribution of effects was found to be significantly heterogeneous (*χ*^2^ (15) = 56.34, *p* < .05), and about three-quarters of that variability is probably due to systematic factors, rather than to merely to random ones (*I*^2^ = 73.4%). Such is a clear analytic invitation to examine potentially important effect moderators such as gender.

**Figure 3:**
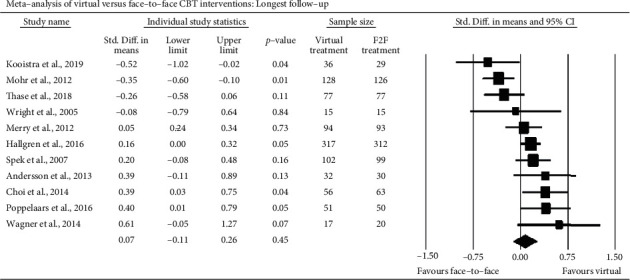
Critical follow-up comparisons of virtual and F2F CBT.

The 11 effects at follow-up are displayed in Figure 3. They seemed close replicates of those at posttest. Ranging from ds of -0.52 to 0.61, such extreme estimates were again associated with the smallest trials. Six of the 11 RCT findings were null. And again, the synthetic estimate at follow-up was in systematic support of the noninferiority hypothesis; *d*_pooled_ = 0.07 (95% CI -0.11, 0.26). Like at posttest, the distribution of effects at follow-up was heterogeneous (*χ*^2^ (10) = 32.76, *p* < .05) and probably mostly due to systematic factors (*I*^2^ = 69.4%).

#### 3.2.2. Moderation by Gender

First, none of the trials tested the noninferiority hypothesis separately by gender. Second, though only one of the selected RCTs had a majority of male participants, it provided a more qualitative finding of suggestive interest [56]. In tentative support of the gender moderator hypothesis, it observed an apparently much greater preexperimental depressive symptom alleviation rate among the virtual (85.3%) than among the F2F CBT (57.3%) study group. However, with samples of only 14 participants in each study group, both its preexperimental and experimental findings were statistically nonsignificant, probably for their lack of power. Next, we recoded study male representation at four different criterion cut-offs, creating four categorical measures of male representation (less than 20% vs. 20% or more boys/men, with similar cuts at 33%, 35%, and 37%). None of these gender proxy measures significantly moderated outcomes at posttest or follow-up.

Finally, in exhaustively exploring possible moderations by other personal, contextual, research design or intervention characteristics, we discovered a meta-analytic interaction that shed some more light on the potential gender divide. We first found that unblended, purely virtual CBT programs produced larger effects (*d*_pooled_ = 0.17 [95% CI -0.02, 0.37]) than blended ones (*d*_pooled_ = −0.30 [95% CI -0.55, -0.05]). Then, we found a pattern only at follow-up suggesting that such relatively larger effects (i.e., relatively larger impacts of virtual versus F2F CBT) were consistently observed among studies with greater male representation (see Table 2).

We also tested professional guidance as a moderator by separating studies into whether virtual trials utilized self-help sessions or offered any level of professional guidance (some professional guidance or all sessions completed with a professional), but the results were null at both posttest measurement and longest follow-up.

As a last note, further explorations only suggested one more moderation, again at follow-up, that is, racialized group status. Trials with sizeable proportions of racialized, ethnic, or cultural minority group members (20% or more;*d* = −0.13[95% CI -0.42, 0.16]) were compared to others (*d* = 0.21[95% CI 0.09, 0.33];*χ*^2^ (1) = 4.55,*p* = .03), suggesting very generally that some members of certain racialized minority group(s) may do better with F2F CBT. Perhaps, virtual environments, technologically or otherwise, relatively disadvantage minoritized people. Or perhaps in contrast to more isolated virtual contexts, F2F ones provide a level of proximity and intimacy and therefore community, which may hold greater value in collectivist cultures associated with certain racialized groups.

## 4. Discussion

With interest greatly potentiated by the COVID-19 pandemic, this meta-analytic study synthesized the best available evidence on the relative effectiveness of virtual versus F2F CBT for working with people who are clinically or subclinically depressed. The potential moderating influence of gender was also explored. Specifically, the notion that men might preferentially benefit from pandemic-related expansions of Internet-based or related telehealth methods of providing mental health services was explored. First, the hypothesized noninferiority of virtual CBT was strongly supported. Twenty of 27 outcomes of the 17 RCTs critically reviewed were null; that is, they found no significant differences between virtual and F2F CBT study groups at either posttest or longer-term, most typically 6 month, follow-up. Moreover, the sample-weighted, pooled standardized mean differences or effect sizes at posttest and follow-up were both essentially zero, bounded by quite narrow confidence intervals. This synthetic finding is important, especially within the current contexts of the ongoing COVID-19 pandemic. The many organizations that, of necessity, shifted much of their service provisions to virtual platforms in order to keep people safe may be comforted by these confidence-inspiring findings. Virtual CBT seems robustly as effective as traditional F2F CBT in alleviating depression, clinical or subclinical, among children to older adults. Such findings will likely remain relevant into the pandemic's uncertain aftermath, but also in analogous circumstances where depressed people are otherwise isolated, geographically (e.g., living in remote places) or socially (e.g., members of marginalized communities). Finally, all such review findings are probably best generalizable to the English-speaking, global west or north, where all of the primary studies were accomplished.

On the other hand, little direct support for the gender moderator hypothesis was found. While the effect distributions were significantly heterogeneous at posttest and follow-up, suggesting that study characteristics (e.g., participants, contexts, research designs, or interventions) can probably ultimately explain them, there was not enough meta-analytic power to test effect moderations by gender. The central problem was that none of the primary studies, the RCTs themselves, tested the noninferiority hypothesis separately for men and women. Future studies ought to do so. Though not directly hypothetically supportive, a number of quite interesting and important descriptive trends related to gender were uncovered. For example, the gross underrepresentation of men in this field's trials seems of profoundly practical and scholarly importance. Also, the scant preexperimental and qualitative experiences of 28 participants in one RCT suggested that men may do better with virtual CBT. And finally, using a review generated moderator (proportion of study participants who were boys or men), a few studies suggested again that men may do better in purely (i.e., unblended) virtual treatment environments. In short, the exploration of gender in this field provided some hope but leaves more questions unanswered than answered. The central question though, about the potentially more effective provision of mental health care to depressed men through the Internet or other virtual offerings, essentially remains for future research testing. Another unanswered question seems more practical, even rhetorical, for consideration by those designing this field's trials. If men are so much more likely to die by suicide than women, they may prefer virtual care, and there seem suggestions that virtual care may even be more effective for them. Why are they clearly not being adequately recruited for clinical trials [30, 65]? Such seems an important question for decision-makers and future researchers alike to consider.

Finally, our null finding that virtual CBT was equally effective to F2F CBT regardless of whether the sessions were made up of self-help or professionally guided modules was an interesting finding. The idea that self-help sessions may be equally effective for some individuals as F2F CBT has potential to save money for organizations and increase therapy accessibility for individuals who have inflexible schedules or people who are facing personal barriers that make them less willing to engage in the vulnerability that is often required in a therapeutic relationship. This is supported by Seward and Harris' findings that men reported less willingness to reach out for help when suicidality increased; while Seward and Harris' study focused specifically on men, we believe this finding should be considered in light of all genders.

In the process of conducting our systematic review, we became aware of a similar meta-analysis that compared the efficacy of virtual and F2F CBT for depression [66, 67]. Our meta-analysis updated and extended Luo et al.'s [67] findings by including a comparison at both posttest and longest follow-up, including a unique exploration of moderating factors with special attention to gendered discrepancies in mental health outcomes, and providing several important directions for future research in the field. Importantly, our findings provide additional support for the noninferiority hypothesis; virtual CBT appears to be equally as effective as traditional F2F CBT.

## 5. Further Limitations and Future Research

### 5.1. Primary Studies

While the aggregated meta-analytic study sample was large (*n* = 2,292 participants), many of the primary studies seemed underpowered. Recall that the most typical RCT had 101 participants, so more than half of the RCTs had less than 100 participants. And only two of the trials had more than 100 participants in each of their virtual and F2F study groups. Such relatively small samples may increase the potential for confounding. Future noninferiority trials in this field ought to be powered by samples sufficient to allow the confident detection of quite small between-group differences (e.g., *d* = 0.20; [42]). Also, noninferiority trials may require more power (and larger samples) than traditional, superiority trials as one essentially wants confidence in either a significant or null finding by minimizing both type 1 and type 2 errors. Using standard statistical criteria with noninferiority considerations (1-tailed *α* = 0.05; and power_1−*β*_ = 0.80 or .95), studies in this field may require approximately 600 to 1,100 participants to detect between-group differences characterized by a *d* of 0.20 [46, 48]. Future researchers ought to consider this, especially as they endeavor to develop more confident understandings about subsamples of men, women, nonbinary genders, specific racialized group members, and others.

Additionally, this field's RCT reports often did not provide enough detail about such important information as racialized/ethnic minority group memberships and related indicators of socioeconomic vulnerability such as low-income status or living in poverty, low educational achievement or dropping out of high school, or low occupational prestige. And none of them has yet specifically studied the experiences of any such potentially vulnerable, racialized, or socioeconomic group members. In the era of COVID-19, when stunning race and class-based, health inequities and injustices have been clarified for the entire world, such seem particularly glaring and egregious knowledge gaps [2, 69–72]. Along with this, it is also important to remember the geographical distribution of the articles in the sample. All the research reviewed came from developed, high-income countries. It is possible that our search criteria influenced this finding; however, as a result of this, our findings may not be generalizable on a global scale. This limitation, along with our finding of heterogeneity in long-term follow-up measurements, suggests the need for a full, multilingual systematic review. Such a full-scale review could provide valuable insight into the scientific production surrounding this topic in other geographical regions (e.g., the global south) and create a more holistic perspective of the research on the comparative efficacy of face-to-face and virtual CBT for depression on a global scale.

Resounding previous reviewers, we recommend that every effort be made with future, more powerful trials to collect more detailed demographic and socioeconomic data and to use it in planning more confident comparisons, not only between men and women but also between the members of specific racialized groups, and ultimately to examine the probably most interesting and important intersections of gender and race and class [73–76].

### 5.2. Rapid Review and Meta-Analysis

As with all rapid reviews, this one was subject to certain fiscal and temporal constraints. Consequently, we could not follow every single PRISMA recommendation [40, 41]. For our lack of library science resources, software, and human, we could not unduplicate our research literature searches and were not blind to the primary studies' findings. However, each step of the review process, selection of studies, data extraction, and meta-analysis was reliably cross-validated by two reviewers. Therefore, we believe that despite its rapid nature, our rapid review findings could be systematically replicated by a full systematic review; in fact, we believe that it should be. Soon this field could benefit from a much better endowed, full systematic review. A series of such more exhaustive, extensive, and complex research syntheses, each perhaps focusing its meta-analytic component on theoretically and practically important moderators such as gender, race, and class, would be most welcome. Such would go a long way toward effectively informing the most relevant mental health care and related decision-makers post-COVID-19.

Finally, one ought to always consider the possibility that publication bias might be a confound explanation for the findings of any review. Though this rapid review and meta-analysis' sampling frame included unpublished, grey research literature sources, ultimately its sample did not include any such so-called grey study reports. Despite this, we think publication bias highly unlikely in this instance for the following reasons. First, critical effects reported in the published RCTs, that is, their standardized mean differences or Cohen's at posttest or follow-up, ranged quite widely (*d*s of -2.33 to 1.11), with 20 of 27 of the findings being null. The field's editors seemed quite open to publishing null even counterhypothetical findings. In fact, publication bias concerns may be relatively moot with noninferiority trials. As they essentially hypothesize the null, their null findings, in a sense, correspond to “significant” results. Second, this review's moderator hypothesis on gender was not the primary hypothetical concern of any of its included studies, so it seems unlike to have been affected by such editorial decisions, whether made by authors or editors.

Still, a future well-endowed, full systematic review might consider expanding its grey literature sampling frame. We think it is still valuable to represent the diverse voices of the field's diverse knowledge users: scholars, practitioners, decision-makers, and publics. Incidentally, we think that the updated raw material for such a systematic review and meta-analysis will exist soon. Serendipitously, as we searched for primary RCTs, we informally found dozens of potentially relevant RCT protocols that seemed ongoing during the COVID-19 pandemic and highly relevant to the social inequities and injustices it is helping to clarify. A timely synthesis of this evidence may be critical for future pandemic preparers as well as health care reformers. Finally, to ensure the worldwide clinical and policy utility of such a full, well-endowed systematic review, its sampling frame ought to incorporate primary RCT reports from the global east and south written in languages other than English. Assuming that primary investigators will have heeded the call to test differential effects by gender, such a future systematic review ought to test the moderation of overall effects by gender.

## 6. Conclusion

This rapid review and meta-analysis synthesized the best available evidence on the relative effectiveness of virtual versus F2F CBT for people with significant symptoms or diagnoses of depression. With near unanimity, 17 RCTs supported the noninferiority of virtual CBT provided via the Internet or telephone. This and related knowledge will be of clear policy significance well into the pandemic's aftermath. Some indirect evidence suggested moderation by gender that depressed boys and men may, in fact, do better with virtual CBT. However, there was insufficient meta-analytic power to test this gender hypothesis directly. Relatedly, this field's most telling descriptive finding was that boys/men have been grossly underrepresented in its trials. Future trials ought to heed COVID-19's warnings, oversampling those who have been at this field's margins. This ought to advance this field's next generation of knowledge, allowing us to best serve men (and women), those who live in poverty, Indigenous, Black, and other People of Colour, as well as any others at risk of being marginalized or oppressed in contemporary mental health care systems.

## Figures and Tables

**Table 1:** Description of 17 studies included in the meta-analysis with within-group improvement rates at posttest.

Citation Country	Sample characteristics	Depression measures	Intervention group	Analytic samples ITT/PP	Online *d* (95% CI) *U*_3_
			Control group		Offline *d* (95% CI) *U*_3_
			Intensity and duration		
[48] Sweden	Adults, ages 18 or older, and M = 42	BDI	Internet-based CBT	32	1.51 (1.19, 1.83) 93.4%
	22% male	HDRS	Group face-to-face CBT	33	1.21 (0.87, 1.55) 88.7%
	Major depressive disorder	MADRS-S	7-8 weekly sessions	PP	
[49] USA	Homebound, ages 50 or older, and M = 65		Telephone PST	56	1.42 (1.01, 1.83) 92.2%
	22% male, 42% AA, and 25% Hispanic	HAMD	Face-to-face PST	63	1.95 (1.53, 2.37) 97.4%
	HAMD ≥ 15		6 weekly sessions	ITT	
[50] USA	Dementia caregivers, 18 or older, and M = 58	PHQ-9	Telephone CBT ^a^	6	1.46 (0.19, 2.73) 92.7%
	9% male, 100% AA		Face-to-face CBT ^a^	5	0.43 (-0.77, 1.63) 66.6%
	At least moderately depressed		12 weekly sessions	PP	
[51] Sweden	Adults, ages 18 to 71, and M = 43	MADRS	Internet-based CBT	317	1.50 (1.32, 1.68) 93.3%
	25% male		Face-to-face CBT	312	0.86 (0.70, 1.02) 80.5%
	PHQ − 9 ≥ 10		12 weekly sessions	ITT	
[52] USA	Primary care patients, ages 18 or older	PHQ-9	Telephone CBT	87	n.d.
	18% male, 33% AA, and 32% Latinx	HSCL-20	Face-to-face CBT	84	n.d.
	Major depressive disorder		6-8 sessions ^b^	ITT	
[53] Netherlands	Adult outpatients, M = 39	IDS-SR_30_	Blended CBT ^c^	35	1.03 (0.58, 1.48) 84.7%
	37% male		Face-to-face CBT	30	1.05 (0.54, 1.56) 85.3%
	Major depressive disorder		15-20 weekly sessions	PP	
[54] New Zealand	Adolescents, ages 12 to 19, and M = 16	CDRS-R	Computerised CBT ^d^	94	0.75 (0.54, 0.95) 77.3%
	34% male, 24% Mãori	RADS-2	Face-to-face CBT	93	0.62 (0.41, 0.83) 73.2%
	Depressive symptoms		7 modules over 4-7 weeks	ITT	
[55] USA	Primary care patients, 18 or older, and M = 48	HAMD	Telephone CBT	150	1.73 (1.55, 1.91) 95.8%
	22% male, 22% AA, and 16% Hispanic	PHQ-9	Face-to-face CBT	136	1.64 (1.45, 1.83) 94.9%
	Major depressive disorder		18 sessions over 12 weeks	PP	
[56] USA	Children, ages 8 to 14, and M = 10	CDI	Videoconferencing CBT	14	1.05 (0.26, 1.84) 85.3%
	71% male, 21% Hispanic		Face-to-face CBT	14	0.19 (-0.55, 0.93) 57.5%
	Childhood depression		8 weekly sessions	ITT	
[57] Netherlands	Adolescents, ages 11 to 16, and M = 13	RADS-2	Computerised CBT ^d^	51	1.09 (0.67, 1.51) 86.2%
	0% male		School-based CBT program ^e^	50	0.75 (0.34, 1.16) 77.3%
	Depressive symptoms		7-8 weekly sessions	ITT	
[58] Australia	University students, ages 18 to 23, and M = 19	DASS-21	Online CBT ^f^	9	0.10 (-0.82, 1.02) 54.0%
	26% male		Face-to-face CBT	10	2.88 (1.63, 4.13) 99.8%
	Depressive symptoms		5 sessions over 3 weeks	ITT	
[59] Australia	Young adults, ages 18 to 25, and M = 20	DASS-21	Online CBT ^f^	23	1.34 (0.70, 1.98) 90.9%
	27% male, 23% Middle Eastern		Face-to-face CBT	21	2.75 (1.91, 3.59) 99.7%
	Depressive symptoms		5 weekly sessions	ITT	
[60] Netherlands	Older adults ages, 50 to 75, and M = 55	BDI-II	Online CBT	67	1.05 (0.72, 1.38) 85.3%
	35% male		In-person group-based CBT ^g^	63	0.66 (0.34, 0.98) 74.5%
	Subthreshold depression		8 or 10 weeks, respectively	PP	
[61] Netherlands	Older adults, ages 50 to 75, and M = 55	BDI-II	Online CBT	102	
	37% male		In-person group-based CBT ^g^	99	n.a.
	Subthreshold depression		8 or 10 weeks, respectively	ITT	
[62] USA	Medication-free adults, M = 46	HAMD	Computer-assisted CBT ^h^	77	2.55 (2.30, 2.80) 99.4%
	34% male, 21% AA, and 7% Hispanic	IDS-SR_30_	Face-to-face CBT	77	2.20 (1.97, 2.43) 98.6%
	Major depressive disorder	BDI-II	20 sessions over 16 weeks	ITT	
[63] Switzerland	Adults, ages 19 to 67, and M = 38	BDI-II	Therapist-guided online CBT	25	1.31 (0.73, 1.89) 90.5%
	35% male		Face-to-face CBT	28	1.35 (0.79, 1.91) 91.1%
	Depressive symptoms		8 weekly sessions	PP	
[64] USA	Medication-free adult ages 18-65, and M = 40	HAMD	Computer-assisted CBT	15	1.67 (0.84, 2.50) 95.2%
	27% male	BDI-II	Face-to-face CBT	15	1.69 (0.86, 2.52) 95.4%
	Major depressive disorder		9 sessions over 8 weeks	ITT	
Meta-analytic statistics:					
Within virtual intervention group sample-weighted *d*_pooled_ (95% CI) *U*_3_					1.35 (1.25, 1.45) 91.1%
Within F2F control group sample-weighted *d*_pooled_ (95% CI) *U*_3_					1.13 (1.03, 1.23) 87.0%

*Note.* AA: African American; CBT: cognitive behavior therapy; BDI: Beck Depression Inventory; BDI-II: Beck Depression Inventory—Second Edition; CDI: Children's Depression Inventory; CDRS-R: Children's Depression Rating Scale-Revised; CI: confidence interval; DASS-21: Depression Anxiety Distress Scales; HAMD: Hamilton Rating Scale for Depression; HRSD: Hamilton Depression Rating Scale; HSCL-20: Hopkins Symptom Checklist; IDS-SR: Inventory of Depressive Symptomology-Self Report; ITT: intention to treat; K10: Kessler Psychological Distress Scale; M: mean age; MADRS: Montgomery-Åsberg Depression Rating Scale; MDD: major depressive disorder; n.d.: no data available; n.a.: not applicable (only one-year follow-up results included); PP: per protocol; PHQ-9: Patient Health Questionnaire 9-Item; PST: problem-solving therapy; RADS-2: Reynold's Adolescent Depression Scaled-Second edition. ^a^Problem-solving therapy is grounded in CBT. ^b^First four weekly sessions followed by 2 to 4 biweekly sessions. ^c^Ten weekly face-to-face sessions and 9 web-based sessions. ^d^SPARX (smart, positive, active, realistic, X-factor thoughts) interactive fantasy game designed to deliver CBT. ^e^Op Volle Krackt is a school-based CBT program for reducing and preventing depressive symptoms. ^f^Mood GYM computer-based self-help. ^g^Coping with depression 8-week Internet-based self-help course. ^h^Good days ahead multimedia program consisting of 9 Internet-delivered modules and 12 sessions with a therapist.

**Table 2:** Blended versus face-to-face CBT interventions at follow-up measurement.

	Study comparison with face-to-face CBT				
	Blended CBT		Exclusively virtual CBT		
% male	*Nstudies*	*d(CI)*	*Nstudies*	*d(CI)*	Between-group difference
< 20%	1	n.d.	n.d.	n.d.	n.d.
> 20%	3	-0.30 (-0.55, -0.05)	7	0.15 (-0.06, 0.36)	*χ* ^2^ (1) = 7.27, *p* = .007^∗^
< 33%	1	-0.08 (-0.79, 0.64)	5	0.17 (-0.13, 0.46)	*χ* ^2^ (1) = 0.38, *p* = .054
> 33%	2	-0.34 (-0.60, -0.07)	3	0.18 (-0.04, 0.40)	*χ* ^2^ (1) = 8.45, *p* = .004^∗^
< 35%	2	-0.23 (-0.52, 0.06)	6	0.14 (-0.10, 0.38)	*χ* ^2^ (1) = 3.75, *p* = .053
> 35%	1	-0.52 (-1.02, -0.02)	2	0.29 (-0.04, 0.62)	*χ* ^2^ (1) = 7.04, *p* = .008^∗^
< 37%	2	-0.23 (-0.52, 0.06)	7	0.02 (-0.05, 0.41)	*χ* ^2^ (1) = 4.64, *p* = .039^∗^
> 37%	1	-0.52 (-1.02, -0.02)	1	0.20 (-0.08, 0.48)	*χ* ^2^ (1) = 6.17, *p* = .013^∗^
					

Note. CI: confidence intervals; n.d.: no data available. ^∗^p < .05.
